# Silver Nanopartical over AuFON Substrate for Enhanced Raman Readout and Their Application in Pesticide Monitoring

**DOI:** 10.3390/molecules20046299

**Published:** 2015-04-09

**Authors:** Kun Guo, Rui Xiao, Xiaoye Zhang, Chaoguang Wang, Qiqi Liu, Zhen Rong, Lin Ye, Suhong Chen

**Affiliations:** 1 Beijing Key Laboratory of New Molecular Diagnosis Technologies for Infectious Diseases, Beijing Institute of Radiation Medicine, Beijing 100850, China; E-Mails: momokunlun@foxmail.com (K.G.); ruix203@sina.com (R.X.); liuqiqi@bmi.ac.cn (Q.L.); rongzhen0525@sina.com (Z.R.); 2 Department of Occupational and Environmental Health, School of Public Health, Jilin University, Changchun 130021, China; 3 School of Pharmaceutical Sciences, Jilin University, Changchun 130021, China; E-Mail: zxyxiaoye@163.com; 4 College of Mechatronics Engineering and Automation, National University of Defense Technology, Changsha 410073, China; E-Mail: cg.king@163.com

**Keywords:** SERS, Ag NPs, Au film over nanosphere, hybrid substrate, trace chemical detection

## Abstract

Surface-enhanced Raman detection of thiram is demonstrated by using Ag-nanoparticles (Ag NPs) on Au film over nanosphere (AuFON) substrate as the hybrid substrate. The SERS signal of the Ag NPs attached to solid supports is studied. The close coupling together of thousands of Ag NPs on AuFON leads to the generation of hot spots for SERS. The Ag NPs on AuFON can be applied to detect rhodamine-6G (R6G) with the detection limitation of 10^−11^ M and the pesticide thiram in acetone with a detection limit of as low as 0.24 ppm, which is much lower than the maximal residue limit (MRL) of 7 ppm in fruit prescribed by the U.S. Environmental Protection Agency (EPA). The hybrid substrates are shown to be highly sensitive for the detection of thriam, which produce highly enhanced Raman signals with good uniformity and reproducibility due to having plenty of hot spots on its surface.

## 1. Introduction

Surface-enhanced Raman scattering (SERS) technique is one of the most powerful analytical tools for chemical and biological detection due to its high sensitivity and specificity. Due to the advantages of providing vibrational spectroscopic finger prints and acquiring nondestructive signals [1], SERS has been widely used to detect a wide range of targets, including DNA [2,3], proteins [4,5], pesticides [6] and metabolites [7]. However, many approaches of fabricated the SERS substrate have some restrictions and shortcomings which limit their extensive application, such as long processing time, unique special equipment, or low through put. Therefore, formation of nanostructures to solve the poor reproducibility of the SERS-active sites remains a challenge in this field.

A unique SERS platform consisting of a metal film over close-packed nanosphere substrate was first developed in 1984 [8]. Metal film over nanosphere (MFON) are created by vapor deposition of thin films of either silver or gold over spheres (polystyrene or silica) and are optimal SERS substrates in that they are easily made, cost effect, and extremely reproducible over a large area. Reproducible and robust structures that strongly enhance the electromagnetic field are most desirable for SERS [9]. Van Duyne [10] and Fang [11] proposed metal films over nanosphere (MFON) electrodes as SERS active substrates in order to improve the surface nanostructure stability and suppress the inherent loss, where nanocavities with hot-spots are presented [12].

The “hot spots” which contribute to the enhancement of the Raman signal were usually located at the gaps and the corners of noble metal. Yang’s group have shown that narrow interparticle gaps induce “hot spots” that provide a giant electromagnetic SERS enhancement [13]. Up to now, most of the SERS platforms are noble metal colloidal nanoparticles (NPs) where enormous Raman enhancement factors could be obtained in areas of “hot spots” from the locations of colloidal aggregations [14,15,16]. Researchers have studied the effect of the size, shape, and composition of the metal NPs on the SERS signal with the metal NPs dissolved in solution with the analyte or with the metal NPs attached to solid supports [17]. Herein, we report a structure composed of Ag NPs on Au film over nanosphere (AuFON) for enhanced Raman signal readout. Ag nanoparticles can be immobilized on AuFON using aminosilane linkers. This is based on the fact that aliphatic amines can act as reductants and stabilizers simultaneously when preparing Ag nanoparticles by thermal activation [18]. The Ag NPs on AuFON structure was used as the SERS-active substrate to detect rhodamine 6G (R6G). In our group’s previous work, we used the Ag NPs/Au FON detect melamine [19] and in this work we investigate the Raman enhancement behaviors of silver nanoparticles (Ag NPs) with AuFON for the identification and detection of pesticide residues that caused intense public concerns by directly threatening human/animal’s health due to their residues in agricultural products. Thiram (bis-(dimethyldithiocarbamoyl) disulfide), a dithiocarbamate fungicide, is used in agriculture as a fungicide and animal repellent. Moreover, the as fabricated SERS substrate can be used to enhance the Raman signals of thiram, showing a detection limit as low as 0.24 ppb, which is much lower than the maximal residue limit (MRL) of 7 ppm in fruit prescribed by the U.S. Environmental Protection Agency (EPA) [20].

## 2. Results and Discussion

### 2.1. Fabrication and Characterization of the AgNPs/AuFON Structure

**Figure 1:**
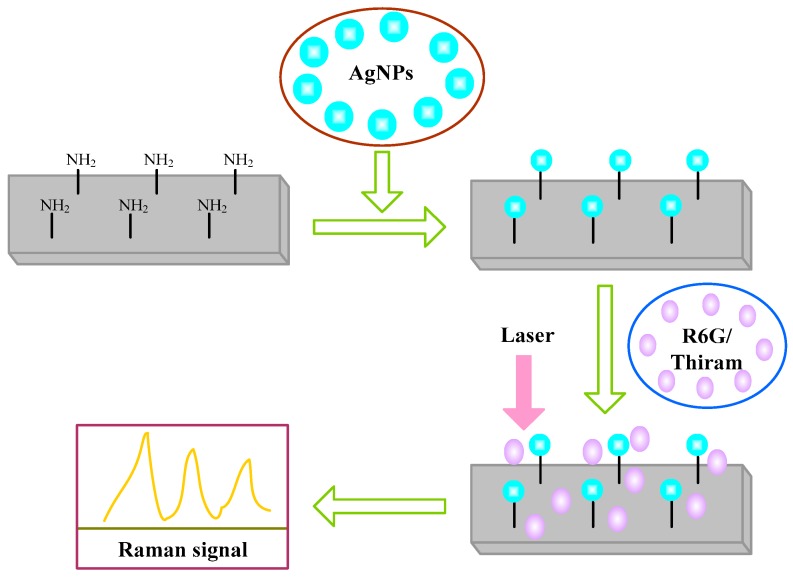
A schematic illustration of the fabrication process of the hybrid substrate and its application in surface-enhanced Raman scattering (SERS).

The fabrication procedure of the hybrid substrate is schematically illustrated in Figure 1. Then the R6G were diluted in a mixer of water and ethanol (*v*/*v* = 1/1) to control the surface tension and the distribution. A 10 uL droplet of the R6G solution was placed onto the slide and dried at room temperature. The Ag NPs colloid was then dropped on the chemicals and again dried at room temperature.

**Figure 2:**
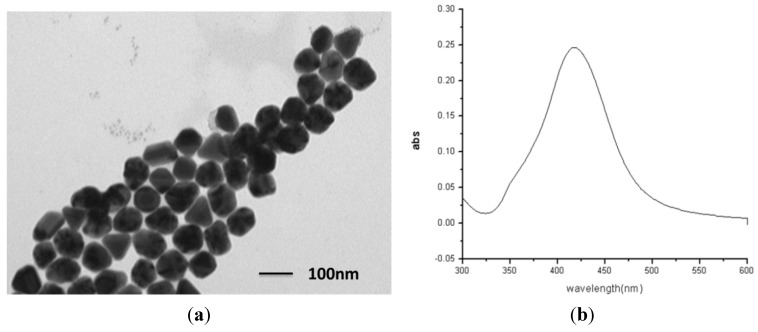
(**a**) Transmission electron microscopy (TEM) images of silver colloidal nanoparticles; (**b**) UV-Vis absorption spectra of silver colloid.

The formation of the Ag NPs was monitored by UV-visible spectroscopy using a SHIMADZU UV-2600 spectrophotometer with 1 nm resolution, and the corresponding surface plasmon absorption in the aqueous solution was observed to be at 426 nm. The shapes and sizes of these Ag particles were better characterized using transmission electron microscope (TEM, Hitachi H-7650, from Japan). Colloidal preparation yields particles that were approximately spherical, as shown in Figure 2a. The average diameter of these SNPs was measured by TEM to be 60 nm. Figure 2a shows TEM images of citrate reduced silver colloidal nanoparticles and Figure 2b shows UV-Vis absorption spectra of silver colloid. With the TEM observation and adsorption spectra, the concentrated colloid can keep stable at a monodispersive state.

**Figure 3:**
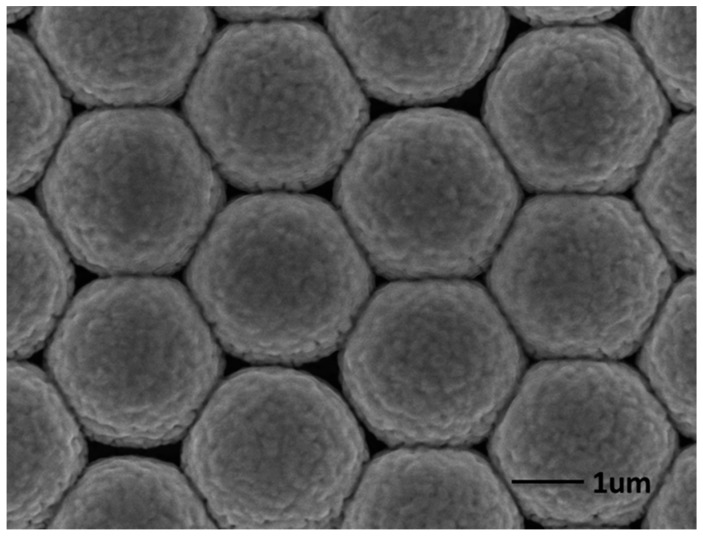
FE-SEM images of the Au film over nanosphere (AuFON) substrate.

**Figure 4:**
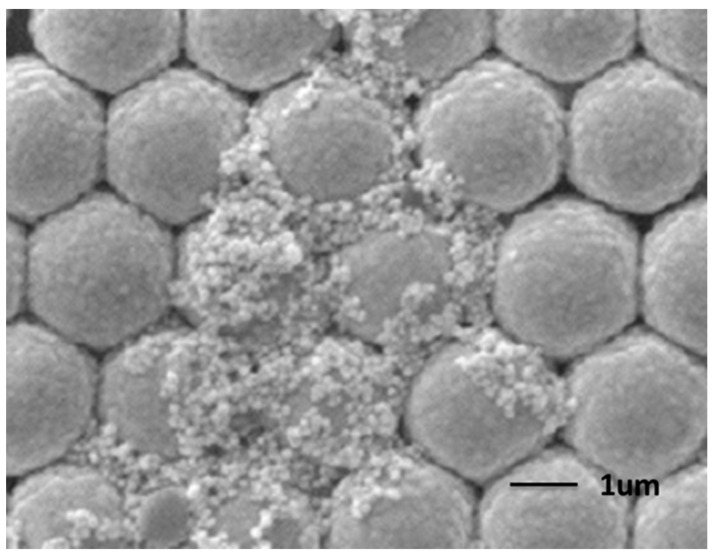
FE-SEM images of the silver nanoparticles (AgNPs)/AuFON substrate.

**Figure 5:**
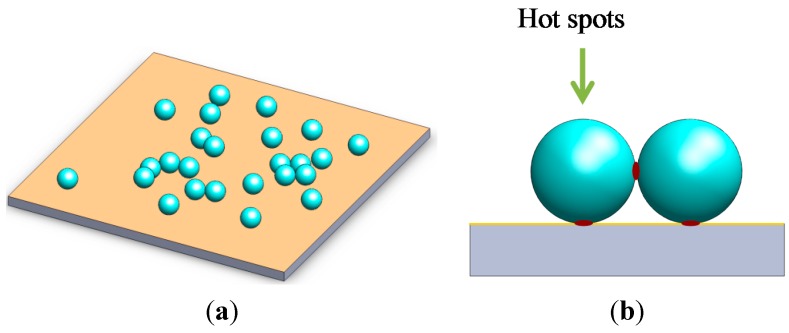
(**a**) The sketch of the hybride substrate; (**b**) shows the location of “hot spots”.

The rough gold surface can be easily observed in Figure 3 and Figure 4, which is essential to SERS. It is well known that the particle aggregation can greatly enhance the Raman signals of target species located at the conjunction of particles by the so-called “hot spots” effect, which is usually achieved by the addition of very concentrated salts. However, this makes the accurate evaluation of the aggregation-based Raman enhancements from colloidal metal NPs almost impossible, due to severe influence on the adsorption of analyte on metal NPs. The as obtained Ag NPs on AuFON substrate can serve as a SERS active substrate for Raman detection with high sensitivity, good reproducibility and high reliability due to having plenty of hot spots on its surface and the unique structure of the hybrid substrate. It has been demonstrated that the “hot spots” which contribute to the enhancement of the Raman signal were usually located at the gaps and the corners of noble metal. The “hot spots” were generated at the conjunctions of the Ag NPs. Ag NPs dropped on the Au film over AuFON covered substrate as shown in Figure 5a. As Figure 5b shows, the “hot spots” of the hybrid substrate are located at not only the conjunctions of the Ag NPs, but also the conjunctions between the Ag NPs and the AuFON.

### 2.2. SERS Properties of the Ag NPs on AuFON as Substrate

**Figure 6:**
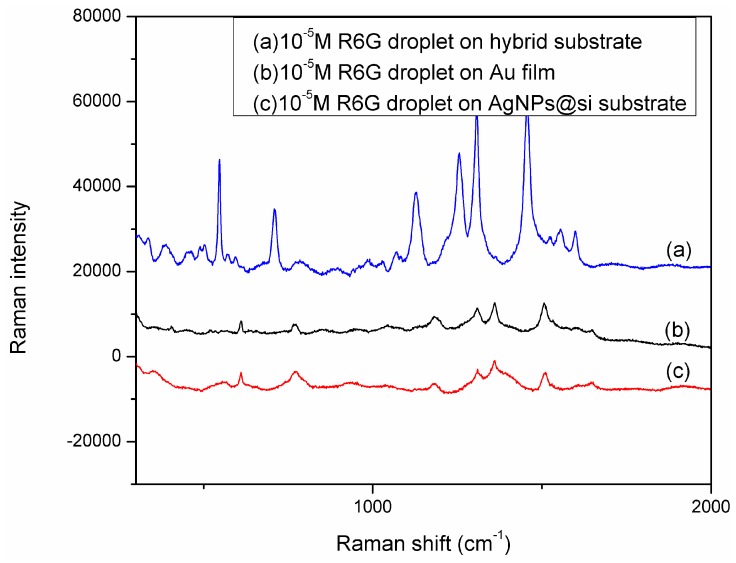
The Raman spectra of R6G measured on different substrates.

R6G was used as a probe molecule to evaluate the SERS performance of the SERS substrate because of its large cross section and well-characterized Raman bands [21]. The strong Raman peaks at 613 cm^−1^, 773 cm^−1^, 1363 cm^−1^, 1510 cm^−1^, and 1651 cm^−1^ are in good agreement with previous reports on pure R6G [22]. From the comparison of relative Raman intensities of each SERS substrate, the substrate containing silver nanoparticles grown for 25–30 min was observed to provide the largest SERS enhancement. Although individual particles exhibit a stronger SERS effect than aggregated ones due to the possible desorption of analytes in the case of particle aggregation, the intensity of Raman signals for the surface detection is dependent on the number of individual particles under one incident laser spot and the amount of target molecules contacted by the particle sensors. Under these conditions, silver nanoparticles with diameters of 40–60 nm were grown inside with amino-terminated silane monolayer on its surface for forming “hot spot” and utilized for further studies. Figure 5 shows (a) 10^−5^ M R6G droplet on hybrid substrate; (b) 10^−5^ R6G droplet on Au film; (c) 10^−5^ M R6G droplet on AgNPs@Si substrate. By comparing Figure 6a–c, it can be easily found that the new SERS substrate was enhanced the Raman intensity comparing with the Au film and Ag NPs @si substrate. Figure 6 shows the Raman spectra of R6G in water with concentrations increasing from 10^−11^ to 10^−7^ M using the hybrid structure as the substrate. The hybrid substrate was sensitive as the detection limitation of the proposed hybrid substrate was 10^−11^ M. The experimental results confirm that the Ag NPs embedded Au film structure has high sensitivity, uniformity, and good reproducibility as the substrate for Raman applications.

### 2.3. SERS Detection of Thiram

**Figure 7:**
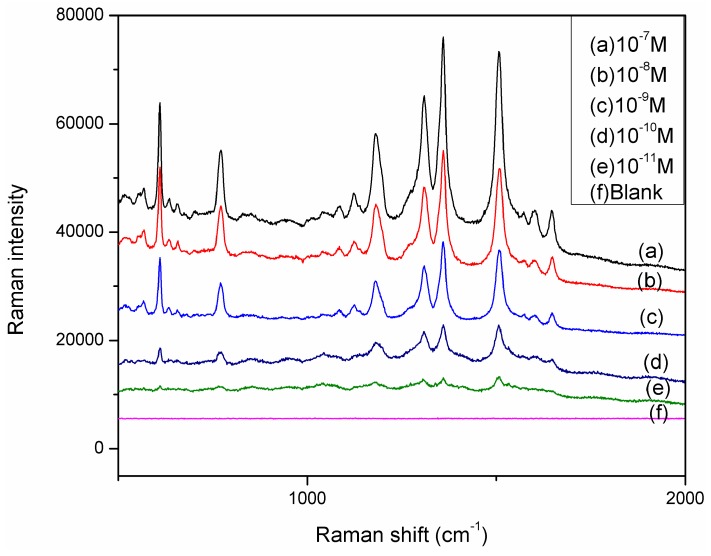
SERS spectra of R6G in water of various concentrations on Ag NPs@AuFON hybrid structure.

**Figure 8:**
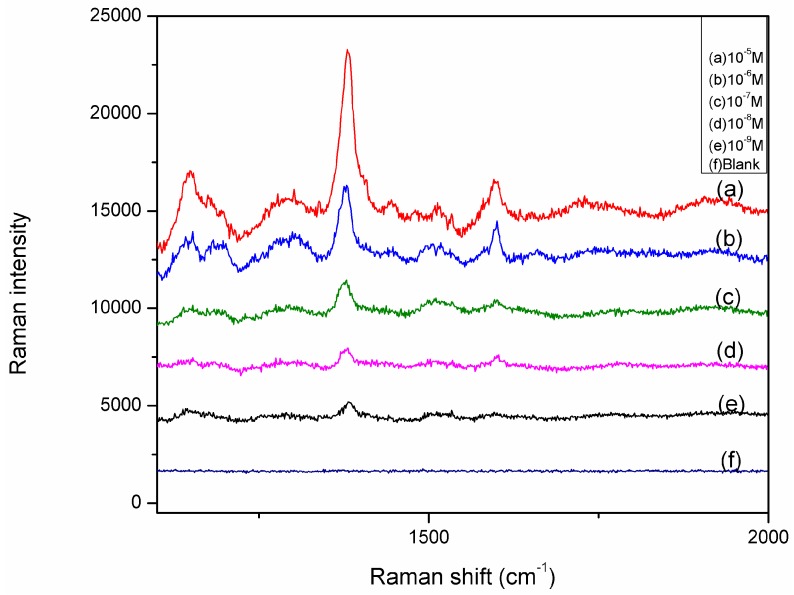
SERS spectra of thiram in acetone of various concentrations on Ag NPs@AuFON hybrid structure.

**Figure 9:**
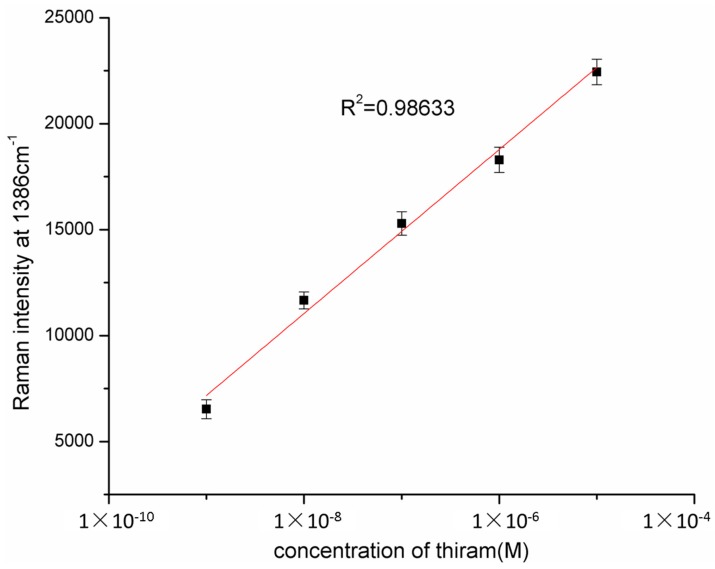
Variations of thiram peak intensity at 1386 cm^−1^ of SERS spectra as a function of thiram concentration.

The hybrid substrate was further applied for the detection of trace thiram, a widely used sulfur-containing pesticide. Which thousands of Ag NPs is closely packed in a cluster leads to the generation of hot spots for SERS. The detection of thiram resulted in a limit of detection (LOD) of 0.24 ppb, which is less than the Environmental Protection Agency prescribed maximum residual (MRL) of 7 ppm in fruit. Figure 7 shows the Raman spectra of thiram in acetone with concentrations increasing from 1.0 × 10^−5^ to 1.0 × 10^−9^ M using the hybride substrate as the substrate. The intensity of Raman peaks of thiram located at 1148 cm^−1^, 1386 cm^−1^, 1507 cm^−1^ increase with the thiram concentration [23]. Figure 8 shows the relationship between SERS intensity at 1386 cm^−1^ and the concentrations of the thiram in acetone. The characteristic Raman peak at 1386 cm^−1^ was monitored for different thiram concentrations. The signal intensity increased concomitantly with an increase in thiram concentration, and the resulting calibration curve (Figure 9) was used for the quantitative determination of thiram. The coefficient of determination was 0.98633. The detection of thiram exhibited a better linear calibration curve [24]. A good linear response was found in the 10^−5^ to 10^−9^ M range. The error bars that represent the standard deviation of the mean values were drawn from three times on different areas of each substrate. The limited error bars revealed the uniformity of the substrate. All the fingerprint SERS signals of thiram could be observed clearly from the five samples. The results imply that the as-fabricated hybrid substrate can be used to detect thiram in all of the different matrices tested with high sensitivity and good reproducibility.

## 3. Experimental Section

### 3.1. Materials and Equipment

R6G, 3-aminopropyltriethoxysilane (APTES), silver nitrate (AgNO_3_), Citric acid trisodium salt dihydrate (C_6_H_5_Na_3_O_7_·2H_2_O), ethanol (C_2_H_5_OH), ethanoic acid was purchased from Sigma-Adrich (St. Louis, MO, USA). Thiram was purchased from Aladdin Chemistry Co. Ltd. (Shanghai, China). Gold (Au) pellets with the purity of 99.999% were purchased from Jinyu Aochen (Beijing, China). Twice distilled water (resistance rate ≥ 18.2 Mw·cm) was utilized as water samples. The SERS spectra were recollected using a portable Raman system (innoRam, B&W Tek, Newark, DE, USA). The 785 nm diode laser was used as excitation light (300 mili-watts of optical power). Transmission electron microscopy (TEM, H-7650, Hitachi, Japan). UV-Vis spectroscopy using a UV-2600 spectrophotometer (SHIMADZU, Kyoto, Japan). FE-SEM micrographs were acquired using a JSM-7001F FE-SEM (JEOL, Tokyo, Japan) operating at 50 kV. 

### 3.2. Silver Colloids Preparation

The synthesis of silver colloids was prepared by using microwave synthesis method [25,26]. First, silver nitrate (37 mg) was suspended in 100 mL ultrapure water, and then 2.6 mL 1% sodium citrate solution was added. After vigorous stirring, the mixed solution was placed in a microwave oven. After several minutes of boiling under high-fire (600 W), the conical flask was removed from the oven and cooled at Room Temperature. After cooling, we adjusted the volume to 100 mL with ultrapure water to ensure consistency between several experiments. The resulting colloid exhibited a dark gray color. The synthesized silver colloids were investigated by means of TEM and UV-Vis absorption spectrum. When measuring the UV-Vis absorption spectrum, the silver colloids were diluted 100 times with ultrapure water.

### 3.3. Fabrication the AgNPs/ AuFON Structure as Hybrid Substrate

The AuFON covered substrate was fabricated with an e-beam evaporator (ZZS500, Nanguang, Chengdu, China). The cleaned Si wafer was horizontally placed in the evaporator. The chromium layer (50 nm) and the gold layer (200 nm) were deposited on the silicon substrate orderly. The Cr film served as an adhesive layer. And the metal deposition process was monitored with the quartz crystal resonator. The prepared AuFON substrate was immerged in the piranha solution (a mixture of concentrated H_2_SO_4_ and 30 wt % H_2_O_2_ at the volume ratio of 4:1) at 80 °C for an hour to improve the Hydrophilicity. Then the wafer was cut into 5 mm × 5 mm slides. The Au films substrate was modified by silanizatio with APTES, resulting in an amino-terminated silane monolayer on its surface for further chemical conjugation with the Ag NPs. Au films were prepared by mixing 50.0 μL ethanol, 0.5 μL 1% (volvol) (3-aminopropyl) triethoxysilane (APTES), 0.05 μL 1‰ ethanoic acid, and after 1 h washed with water three times. Next, the APTES-modified substrate was immersed in a colloidal suspension of Ag NPs, leading to a layer of Ag NPs on the AuFON substrate.

### 3.4. Raman Detection of R6G in Water

Ten-milliliter aliquots of different concentrations of R6G in water were dispersed on the as-prepared substrates and dried in an air for SERS detection. The Raman spectra were recorded using a 785 nm laser with 1 mW power and a 50 × objective (1 μm^2^ spot). The integral time was 5 s and the slit aperture was 10 μm.

### 3.5. Raman Detection Thiram in Spiked Samples

For the detection of thiram in acetone, 10 mL aliquots of different concentrations of thiram in acetone were dispersed on the as-prepared substrates. For the detection of thiram in spiked samples, 100 mL of the spiked sample solution was diluted in ethanol to 1 mL, then 10 mL of the diluted solution was dropped onto the substrate. The Raman spectra were recorded using 785 nm laser with 3 mW power and a 50 × objective (1 μm^2^ spot). The integral time was 10 s and the slit aperture was 10 μm.

## 4. Conclusions

In summary, we have developed Ag NPs on AuFON through a simple self-assembly process as an effective SERS substrate. The Ag NPs on AuFON as SERS substrate could produce highly enhanced Raman signals with good uniformity and reproducibility due to having plenty of hot spots on its surface. The Ag NPs on Au film nanostructure applied to detect the pesticide thiram in acetone with a detection limit as low as 10^−9^ M (0.24 ppm), which is lower than the maximal residue limit (MRL) of 7 ppm in fruit prescribed by U.S. Environmental Protection Agency (EPA). The simple, rapid, and ultrasensitive analytical techniques for the on-site analysis of trace pesticide residues will improve environmental and agricultural safety.
